# Comparison of traction vs. snare as rescue methods for challenging colorectal endoscopic submucosal dissection: Propensity score-matched study

**DOI:** 10.1055/a-2544-3279

**Published:** 2025-03-14

**Authors:** Keitaro Takahashi, Takuya Iwama, Kazuyuki Tanaka, Yuki Miyazawa, Shohei Kuroda, Masashi Horiuchi, Seisuke Saito, Momotaro Muto, Katsuyoshi Ando, Nobuhiro Ueno, Shin Kashima, Kentaro Moriichi, Hiroki Tanabe, Mikihiro Fujiya

**Affiliations:** 1 38051 Division of Gastroenterology, Department of Internal Medicine, Asahikawa Medical University, Asahikawa, Japan; 2 26853 Gastroenterology, Asahikawa City Hospital, Asahikawa, Japan; 3 13741 Gastroenterology, Asahikawa Kosei General Hospital, Asahikawa, Japan; 4 13739 Gastroenterology, Nayoro City General Hospital, Nayoro, Japan; 5 26837 Gastroenterology, Asahikawa Red Cross Hospital, Asahikawa, Japan; 6 26385 Gastroenterology, Sapporo Higashi Tokushukai Hospital, Sapporo, Japan; 7 36802 Gastroenterology, Furano Kyokai Byoin, Furano, Japan; 8 38066 Internal Medicine, Engaru-Kosei General Hospital, Monbetsu-gun, Japan

**Keywords:** Endoscopy Lower GI Tract, Endoscopic resection (polypectomy, ESD, EMRc, ...), Colorectal cancer, Polyps / adenomas / ...

## Abstract

**Background and study aims:**

To address the challenges of difficult colorectal endoscopic submucosal dissection (ESD), conversion to snare resection (rescue-snare ESD: rSnare), a variant of hybrid ESD, is commonly proposed. However, rSnare is associated with a lower en bloc resection rate compared with conventional ESD. Traction-assisted ESD has emerged as a technique to facilitate dissection, but its effectiveness as a rescue method remains unclear. This study was the first to compare the effectiveness of rSnare and rescue-traction-assisted ESD (rTraction).

**Patients and methods:**

This retrospective study involved 1464 consecutive lesions from 1372 patients with superficial colorectal neoplasms across eight institutions. Among these, 162 lesions required rescue methods of rSnare or rTraction. After propensity score matching, 88 lesions treated with either rSnare or rTraction were analyzed.

**Results:**

The rTraction group exhibited significantly higher en bloc resection and R0 resection rates (93.2% and 77.3%, respectively) compared with the rSnare group (45.5% and 38.6%, respectively). However, average procedure time was significantly longer in the rTraction group (122.3 ± 72.5 min) compared with the rSnare group (92.2 ± 54.2 min). In the rTraction group, univariable and multivariable analyses identified traction initiation time > 75 minutes as the only independent predictor of procedure durations exceeding 120 minutes.

**Conclusions:**

Utilizing a traction device as a rescue technique in difficult colorectal ESD resulted in higher en bloc and R0 resection rates compared with conversion to snare resection. Initiating traction within 75 minutes may contribute to reducing overall procedure time for challenging colorectal ESD cases.

## Introduction

Endoscopic submucosal dissection (ESD) for colorectal neoplasms demonstrates a higher en bloc resection rate compared with endoscopic mucosal resection (EMR) 1 . However, colorectal ESD presents technical challenges, often involving difficult scenarios such as large tumors, severe fibrosis, and poor scope operability 2 3 4 5 . To address these challenges, conversion to snare resection, termed rescue-snare ESD (rSnare), a variant of hybrid ESD, has been proposed for colorectal ESD 6 . However, en bloc resection rate with rSnare reportedly ranges from 43.6% to 66.7%, which is lower than that achieved with the full ESD procedure 6 7 . In addition, rSnare is associated with a higher rate of local recurrence and an increased need for surgery compared with full ESD 6 . Therefore, it is crucial to explore alternative rescue methods to improve the en bloc resection rate instead of relying solely on rSnare.

Recently, there have been advancements in traction devices, including the internal magnet traction device, clip-and-thread device, spring-and-loop with clip (S-O clip), and multi-loop traction device (MLTD) 8 9 10 11 . Counter traction provided by these devices enhances visibility of the cutting line, leading to more effective and safer ESD 12 . Several studies have illustrated the effectiveness of accelerating dissection speed through planned traction use compared with conventional ESD 13 14 . However, the utility of traction devices as rescue methods for challenging colorectal ESD remains unclear. Herein, we present the first comparative study assessing effectiveness of rescue-traction-assisted ESD (rTraction) versus rSnare using propensity score matching.

## Patients and methods

### Study patients

From November 2014 to October 2022, this retrospective study included 1464 consecutive lesions from 1372 patients with superficial colorectal neoplasms who underwent ESD at eight institutions: Asahikawa Medical University Hospital, Asahikawa City Hospital, Asahikawa-Kosei General Hospital, Nayoro City General Hospital, Japanese Red Cross Asahikawa Hospital, Sapporo Higashi Tokushukai Hospital, Furano Kyokai Hospital, and Engaru-Kosei General Hospital. Fifty-six lesions were excluded due to incomplete data, resulting in enrollment of 1408 lesions from 1316 patients with colorectal neoplasms. Subsequently, referencing the medical records, we identified 162 lesions requiring rescue methods, with 97 lesions treated using rSnare and 65 lesions treated with rTraction. To distinguish between planned and rescue use of snare or traction, we extracted lesions explicitly documented as experiencing intraoperative difficulty in the medical records. Intraoperative difficulty was defined as encountering obstacles such as severe fibrosis, muscle injury or perforation, deep breathing or hyperperistalsis, poor scope maneuverability, and inadequate visibility of the cutting line 2 3 4 5 . This study underwent a centralized review by the Ethics Committee of Asahikawa Medical University and received approval from each participating institution with the approval number C22102. Informed consent was obtained using an opt-out method for this retrospective study.

### Indication and procedure for rSnare and rTraction

**Fig. 1:**
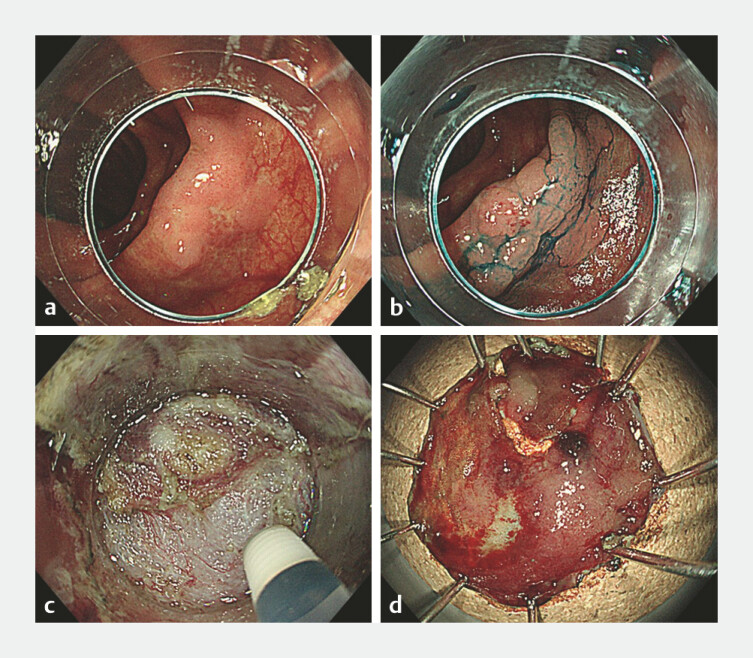
rSnare for a colorectal tumor. **a** A laterally spreading tumor, granular type (LST-G), is identified in the ascending colon. **b** Chromoendoscopy reveals a slightly elevated lesion without signs of invasion. **c** During submucosal dissection, visibility of the cutting line is compromised due to fibrosis. **d** The rSnare technique is performed, resulting in piecemeal resection.

**Fig. 2:**
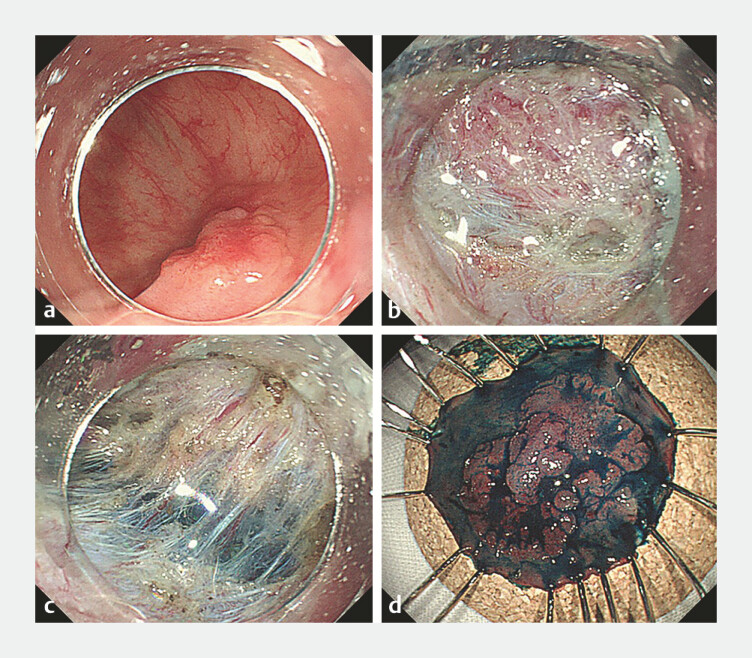
rTraction for a colorectal tumor. **a** A laterally spreading tumor, non-granular type (LST-NG) is detected in the cecum. **b** During submucosal dissection, visibility of the cutting line is poor due to close proximity to the muscle layer. **c** Rescue traction is applied, enhancing visibility of the submucosal layer. **d** En bloc resection is successfully achieved.

ESD procedures were performed either by experts with experience in conducting over 50 ESD procedures or by non-experts under the guidance of experts at each institution. A single-channel lower gastrointestinal endoscope (PCF-H290ZI or PCF-Q260AZI; Olympus Medical Systems, Tokyo, Japan) was utilized along with a high-frequency generator (VIO-300D or VIO3; Erbe Elektromedizin GmbH, Tübingen, Germany). Endoscopists selected an electrosurgical knife from either FlushKnife (DK2620J; Fujifilm Corporation, Tokyo, Japan) or DualKnifeJ (KD-655Q; Olympus Medical Systems, Tokyo, Japan). To lift the mucosa, hyaluronic acid solution (Mucoup; Boston Scientific Corporation, Tokyo, Japan, or K smart; Olympus Medical Systems, Tokyo, Japan) or sodium alginate (Liftal K; Kaigen Pharma Co., Ltd., Osaka, Japan) was injected into the submucosal layer. The ESD process preceding rescue methods followed protocols outlined in the previous reports 15 16 . When endoscopists determined that treating the tumor without a rescue method would be challenging due to intraoperative difficulties, they chose to employ a snare or traction device to address the situation. In the rSnare procedure, the Rotatable Snare (Boston Scientific Corporation, Tokyo, Japan) was utilized following a circumferential incision ( Fig. 1 ). For rTraction, the traction device was selected from an internal traction device (S-O clip; Xeon Medical Inc, Tokyo, Japan, MLTD; Boston Scientific Corporation, Tokyo, Japan, or a handmade device using a thread 17 ). After performing a circumferential incision, the traction device was attached to the anal side of the lesion. Subsequently, with proper traction applied to the lesion, submucosal dissection proceeded from the proximal to the distal side, culminating in en bloc resection ( Fig. 2 ). Traction initiation time was identified based on endoscopic images, videos, or recorded endoscopic findings.

### Statistical analyses

All statistical analyses were performed using R Project for Statistical Computing version 4.0.5 software program. Continuous variables were compared using Student’s *t* -test, whereas Fisher’s exact test was employed for nominal scale data comparison. *P* < 0.05 was considered statistically significant. To minimize potential confounding between the rSnare and rTraction groups, propensity score matching (PSM) was performed using logistic regression with 1:1 nearest neighbor matching. Confounders were identified and adjusted using a Directed Acyclic Graph (DAG) created via DAGitty software. Relative risks (RRs) and 95% confidence intervals (CIs) were calculated to assess associations between variables and outcomes. Variables included in multivariable analysis were selected based on clinical relevance and hypothesized causal relationships, guided by the DAG. Multivariable analysis was conducted using a Poisson regression model. Receiver operating characteristic (ROC) analysis was used to determine optimal cut-off values for tumor size and traction initiation time associated with longer procedure durations. These cut-off values were identified by maximizing the Youden index (sensitivity + specificity − 1). Sensitivity, specificity, and their corresponding CIs were calculated for each potential cut-off point to ensure robustness. The area under the ROC curve (AUC) was calculated to evaluate the discriminatory ability of each variable, with 95% CIs reported. AUC values > 0.5 were considered acceptable, with thresholds of ≥ 0.7 indicating “acceptable” and ≥ 0.8 indicating “excellent” performance.

## Results

### Clinicopathological characteristics of rSnare and rTraction groups

The research flowchart is presented in **Supplementary Fig. 1** . The rate of challenging ESD lesions that required the rescue methods of rSnare and rTraction was 11.5% (162/1408). Among these lesions, 59.9% (97/162) were treated with rSnare and 40.1% (65/162) were treated with rTraction. Clinicopathological characteristics before PSM are summarized in **Supplementary Table 1** . In the rSnare group, rescue methods were required due to fibrosis (16/97, 16.5%), muscle injury (5/97, 5.2%) or perforation (4/97, 4.1%) (total: 9.3%), deep breathing or hyperperistalsis (17/97, 17.5%), poor scope operability (25/97, 25.8%), and poor visibility of the cutting line (30/97, 30.9%). Similarly, in the rTraction group, rescue methods were needed due to fibrosis (14/65, 21.5%), deep breathing or hyperperistalsis (3/65, 4.6%), poor scope operability (23/65, 35.4%), and poor visibility of the cutting line (25/65, 38.5%). Among these factors, incidences of muscle injury or perforation and deep breathing or hyperperistalsis were significantly higher in the rSnare group compared with the rTraction group. Regarding intraoperative perforation, sixd of 97 cases (6.2%) occurred in the rSnare group. Of these, four cases occurred before snaring and were subsequently converted to the rescue snare method, whereas two cases occurred during snaring. In contrast, intraoperative perforation was identified in two of 65 cases (3.1%) in the rTraction group, both of which occurred after traction. Rates of en bloc resection and R0 resection were significantly higher in the rTraction group (95.4% and 81.5%, respectively) compared with the rSnare group (52.6% and 41.2%, respectively). However, background characteristics of the rSnare and rTraction groups differed significantly, in terms of adenoma rate (43.3% vs. 26.2%), tumor size (20.8 ± 8.9 mm vs. 28.3 ± 13.6 mm), and reasons for rescue.

To address these differences, PSM was performed. The model was developed based on a DAG analysis created using existing research findings, incorporating confounders such as lesion characteristics (location, morphology, pathology, fibrosis, submucosal invasion, and tumor size), and technical factors (endoscopist experience and reasons for rescue) ( **Supplementary Fig. 2a** ) 18 19 20 21 . In contrast, patient characteristics (sex and age) were excluded because they were considered not to have a direct influence on treatment allocation or outcomes. The C-statistic of the propensity score model, representing the AUC, was 0.781 (95% CI 0.708–0.854), demonstrating an acceptable level of discriminatory ability. After PSM, 44 lesions treated with rSnare and 44 lesions treated with rTraction were analyzed. Clinicopathological features of the rSnare and rTraction groups are summarized in Table 1 . No significant differences were observed between the two groups in terms of sex, age, expertise level, tumor location, morphology, pathology, fibrosis, invasion depth, tumor size, or reasons for rescue. En bloc resection and R0 resection rates were significantly higher in the rTraction group (93.2% and 77.3%, respectively) compared with the rSnare group (45.5% and 38.6%, respectively). Average procedure time was significantly longer in the rTraction group (122.3 ± 72.5 min) compared with the rSnare group (92.2 ± 54.2 min). Dissection speed was 5.7 mm ^2^ /min in the rSnare group and 4.5 ± 3.0 mm ^2^ /min in the rTraction group ( *P* = 0.12). There were no significant differences in complications such as perforation and post-ESD bleeding.

**Table:** **Table 1** Clinicopathological features of the rSnare and rTraction groups after propensity score matching.

	rSnare	rTraction	P value
Lesions, n	44	44	
Sex, n (%)			0.83
Male	23 (52.3)	25 (56.8)	
Female	21 (47.7)	19 (43.2)	
Age, years, mean (SD)	70.5 (10.8)	69.7 (9.6)	0.72
Expert, n (%)	34 (77.3)	35 (79.5)	0.99
Location, n (%)			0.31
Right side of the colon	31 (70.5)	29 (65.9)	
Left side of the colon	5 (11.4)	10 (22.7)	
Rectum	8 (18.2)	5 (11.4)	
Morphology, n (%)			0.81
Protruded	10 (22.7)	8 (18.2)	
LST-G	10 (22.7)	12 (27.3)	
LST-NG	24 (54.5)	24 (54.5)	
Pathology, n (%)			0.89
adenocarcinoma	29 (65.9)	28 (63.6)	
adenoma	11 (25.0)	13 (29.5)	
SSL	4 (9.1)	3 (6.8)	
Fibrosis, n (%)	13 (29.5)	16 (36.4)	0.65
Invasion depth, n (%)			> 0.99
Submucosal invasion	4 (9.1)	3 (6.8)	
Tumor size, mm, mean, (SD)	22.9 (10.1)	22.8 (8.3)	0.96
Dissection speed, mm ^2^ /min (SD)	5.7 (4.4)	4.5 (3.0)	0.12
En bloc resection, n (%)	20 (45.5)	41 (93.2)	< 0.001
R0 resection, n (%)	17 (38.6)	34 (77.3)	< 0.001
Procedure time, minutes, mean, (SD)	92.2 (54.2)	122.3 (72.5)	0.03
Intraoperative perforation, n (%)	1 (2.3)	2 (4.5)	> 0.99
Post-ESD bleeding, n (%)	3 (6.8)	1 (2.3)	0.62
Reasons for rescue, n (%)			0.14
Fibrosis	10 (22.7)	14 (31.8)	
Muscle injury or perforation	0 (0)	0 (0)	
Deep breathing or hyperperistalsis	7 (15.9)	2 (4.5)	
Poor scope operability	10 (22.7)	16 (36.4)	
Poor visibility of cutting line	17 (38.6)	12 (27.4)	
ESD, endoscopic submucosal dissection; LST-G, laterally spreading tumor, granular type; LST-NG, laterally spreading tumor, nongranular type; SD, standard deviation.			

### Analysis of factors influencing prolonged procedure durations within the rTraction group

**Fig. 3:**
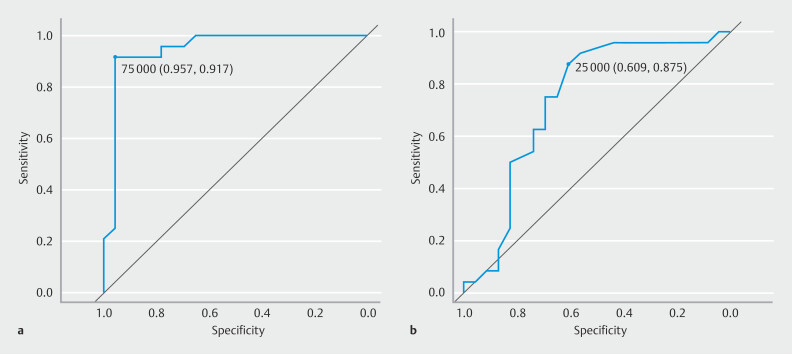
ROC analysis for procedures exceeding 120 minutes. **a** The ROC analysis found that for procedure times exceeding 120 minutes, the threshold for traction initiation time was 75.0 minutes, with an area under the curve (AUC) of 0.915 (95% confidence interval [CI] 0.846–0.984), a sensitivity of 0.917, and a specificity of 0.957. **b** Regarding tumor size associated with procedure times over 120 minutes, the threshold was 25.0 mm, with an AUC of 0.764 (95% CI 0.641–0.887), a sensitivity of 0.875, and a specificity of 0.609.

To investigate factors influencing prolonged procedure durations within the rTraction group, we categorized the rTraction group into those with procedure times exceeding 120 minutes and those with times of 120 minutes or less, based on a previous report highlighting technical challenges of colorectal ESD 22 . Table 2 presents clinicopathological features of the two groups. There were 29 lesions in the ≤ 120-minute group and 36 lesions in the > 120-minute group. No significant differences were found in sex, age, expertise level, tumor location, morphology, pathology, fibrosis, invasion depth, or reasons for rescue between the two groups. En bloc resection and R0 resection rates were comparable between the ≤ 120-minute group (93.1% and 82.8%, respectively) and the > 120-minute group (97.2% and 80.6%, respectively). However, average tumor size was significantly larger in the >120-minute group (32.8 ± 13.8 mm) compared with the ≤ 120-minute group (22.7 ± 11.1 mm). Prevalence of S-O clip usage was significantly higher in the > 120-minute group (86.1%) compared with the ≤ 120-minute group (51.7%). Initiation time for traction was significantly later in the > 120-minute group (116.0 ± 44.7 min) than in the ≤ 120-minute group (46.6 ± 23.0 min). In addition, post-traction procedure time was significantly longer in the > 120-minute group (25.3 ± 19.2 min) compared with the ≤ 120-minute group (82.1 ± 50.2 min). ROC analysis revealed that thresholds for tumor size and traction initiation time associated with procedure times exceeding 120 minutes were 25.0 mm and 75.0 minutes, respectively, with corresponding area under the AUC values of 0.764 (95% CI: 0.641–0.887) and 0.915 (95% CI: 0.846–0.984) ( Fig. 3 **a, b** ).

**Table:** **Table 2** Clinicopathological features of procedure time ≤ 120 minutes and > 120 minute groups in the rTraction group.

	≤ 120 min	> 120 min	P value
Lesions, n	29	36	
Sex, n (%)			> 0.99
Male	18 (62.1)	23 (63.9)	
Female	11 (37.9)	13 (36.1)	
Age, years, mean (SD)	67.4 (12.7)	69.3 (12.1)	0.55
Expert, n (%)	23 (79.3)	22 (61.1)	0.19
Location, n (%)			0.44
Right side of the colon	20 (69.0)	25 (69.4)	
Left side of the colon	4 (13.8)	8 (22.2)	
Rectum	5 (17.2)	3 (8.3)	
Morphology, n (%)			0.13
Protruded	5 (17.2)	5 (13.9)	
LST-G	6 (20.7)	16 (44.4)	
LST-NG	18 (62.1)	15 (41.7)	
Pathology, n (%)			
adenocarcinoma	18 (62.1)	22 (61.1)	0.90
adenoma	8 (27.6)	9 (25.0)	
SSL	3 (10.3)	5 (13.9)	
Fibrosis, n (%)	7 (24.1)	7 (19.4)	0.88
Invasion depth, n (%)			0.32
Submucosal invasion	0 (0)	3 (8.3)	
Tumor size, n (%)	22.7 (11.1)	32.8 (13.8)	< 0.05
Dissection speed, mm2/min (SD)	6.9 (6.3)	5.0 (4.7)	0.167
En bloc resection, n (%)	27 (93.1)	35 (97.2)	0.85
R0 resection, n (%)	24 (82.8)	29 (80.6)	> 0.99
Intraoperative perforation, n (%)	0 (0)	2 (5.6)	0.57
Post-ESD bleeding, n (%)	1 (3.4)	2 (5.6)	> 0.99
Traction device, n (%)			< 0.05
S-O clip	15 (51.7)	31 (86.1)	
MLTD	4 (13.8)	1 (2.8)	
Handmade MLTD	10 (34.5)	4 (11.1)	
Traction initiation time, min, mean (SD)	46.6 (23.0)	116.0 (44.7)	< 0.001
Post-traction procedure time, min, mean, (SD)	25.3 (19.2)	82.1 (50.2)	< 0.001
Reasons for rescue, n (%)			0.09
Fibrosis	10 (34.5)	4 (11.1)	
Muscle injury or perforation	0 (0)	0 (0)	
Deep breathing or hyperperistalsis	1 (3.4)	2 (5.6)	
Poor scope operability	7 (24.1)	16 (44.4)	
Poor visibility of cutting line	11 (37.9)	14 (38.9)	
ESD, endoscopic submucosal dissection; LST-G,laterally spreading tumor, granular type; LST-NG, laterally spreading tumor, nongranular type; MLTD, multi-loop traction device; SD, standard deviation.			

To evaluate factors influencing prolonged procedure times in the rTraction group, univariable and multivariable analyses were performed ( Table 3 and Table 4 ). Factors included in the analysis were selected based on results of the DAG analysis ( **Supplementary Fig. 2b** ) 3 19 23 24 25 26 . Key exposure variables with potential direct effects on procedure time—right-sided colon (tumor location), LST-NG (morphology), fibrosis, tumor size > 25 mm, S-O clip usage for traction, and traction initiation time > 75 minutes—were included to assess their independent contributions. Results of the univariable analysis using relative risk revealed that tumor size > 25 mm (relative risk [RR] 2.91, 95% CI 1.96–4.33; *P* < 0.001), S-O clip usage (RR 2.56, 95% CI 1.33–4.92; *P* < 0.05), and traction initiation time > 75 minutes (RR 4.84, 95% CI 3.16–7.42; *P* < 0.001) were significant factors associated with prolonged procedure times. In multivariable analysis using a Poisson regression model, traction initiation time > 75 minutes was identified as the only statistically significant independent factor (β = 1.18, z = 2.26, 95% CI 1.17–8.96; *P* = 0.02). Other variables, including right-sided colon, LST-NG, fibrosis, tumor size > 25 mm, and S-O clip usage were not statistically significant in this multivariable analysis.

**Table:** **Table 3** Univariable analyses for factors associated with longer procedure time.

	Relative risk	95% CI	P value
Right sided colon	1.01	0.77–1.34	> 0.99
LST-NG	0.69	0.43–1.10	0.14
fibrosis	0.88	0.44–1.75	0.76
Tumor size > 25 mm	2.91	1.96–4.33	< 0.001
S-O clip	2.56	1.33–4.92	< 0.05
Traction initiation time > 75 min	4.84	3.16–7.42	< 0.001
CI, confidence interval; LST-NG,laterally spreading tumor, nongranular type.			

**Table:** **Table 4** Multivariable analyses for factors associated with longer procedure time

	Coefficient (β)	SE	z-value	95% CI	P value
Right-sided colon	–0.04	0.41	–0.10	–0.84, 0.75	0.92
LST-NG	–0.09	0.38	–0.23	–0.83, 0.66	0.82
fibrosis	0.17	0.47	0.35	–0.76, 1.10	0.73
Tumor size > 25 mm	0.69	0.47	1.48	–0.22, 1.60	0.14
S-O clip	0.38	0.55	0.68	–0.71, 1.45	0.50
Traction initiation time > 75 min	1.18	0.52	2.26	0.16, 2.19	0.02
LST-NG, laterally spreading tumor, nongranular type; SE, standard error.					

## Discussion

This is the first report comparing effectiveness of traction devices and snares as rescue methods for challenging colorectal ESD. Despite longer procedure duration, rTraction achieved higher en bloc and R0 resection rates than rSnare. Higher en bloc resection rates have been associated with reduced risk of local recurrence, as well as curative resection being linked to favorable long-term prognosis after colorectal ESD 6 27 . Therefore, rTraction is recommended as the primary rescue method in difficult cases. Regarding rSnare, it resulted in shorter procedure duration but a lower en bloc resection rate. In this study, rSnare was frequently used for lesions complicated by deep breathing, hyperperistalsis, muscle injury, or perforation. In cases of deep breathing or hyperperistalsis, these factors may limit access to the lesion and cause instability during incision. Moreover, managing perforation requires advanced techniques, including additional dissection to create sufficient space for clipping and achieving complete closure with endoclips under challenging conditions 28 . Depending on endoscopist expertise, rSnare may serve as an alternative rescue method, particularly in cases of perforation where minimizing procedure duration is a priority. With respect to lesion characteristics, preoperative indicators of technical difficulty have been reported to include right-sided colon location, LST-NG morphology, larger tumor size, and presence of scarring (fibrosis) 3 18 22 29 30 . In particular, right-sided lesions have been associated with incomplete resection and higher risk of perforation due to poor maneuverability 18 29 31 . Although our study lesions were selected based on intraoperative difficulty, it also included a high proportion of right-sided colon lesions, LST-NG morphology, and fibrosis, suggesting that intraoperative difficulty is closely linked to preoperative risk factors.

Previous studies have demonstrated that ESD with planned snare resection, termed hybrid ESD, requires less procedure time and is simpler to perform than conventional ESD 32 33 34 35 . Planned hybrid ESD has shown a high en bloc resection rate ranging from 82.8% to 94.1%, comparable to conventional ESD 33 34 . However, achieving a high en bloc resection rate with hybrid ESD necessitates specific conditions: Lesion size should be under 20mm, the undetached portion should be less than 15 mm in diameter, and remaining submucosal tissue should not be excessively thick 34 36 . In contrast, rSnare was associated with technical challenges and often failed to meet these criteria, resulting in a lower en bloc resection rate of 45.5% in our study. This lower en bloc resection rate observed in the rSnare group is consistent with findings from previous studies on rescue procedures 6 7 . Conversely, for rTraction, the en bloc resection rate was 93.2%, consistent with the high rates reported in previous studies on non-rescue traction techniques 11 37 . This suggests that completing the ESD procedure using a traction device results in a high rate of en bloc resection even in technically difficult lesions requiring rescue methods.

However, prolonged procedure time associated with rTraction may burden endoscopists and decrease patient tolerance. Conventionally, it has been unclear when to utilize traction as a rescue method to shorten treatment duration. Our results revealed that traction initiation time exceeding 75 minutes was an independent predictor of procedure times exceeding 120 minutes. Therefore, in cases of colorectal ESDs, considering use of rescue methods within 75 minutes from ESD initiation may help complete procedures within 120 minutes. Regarding the traction device, a significantly higher prevalence of S-O clip usage was observed in the > 120-minute group, while no significant difference was observed in multivariable analysis. The > 120-minute group had larger tumors compared with the ≤120-minute group, suggesting that endoscopists tended to choose the S-O clip for larger lesions in this study.

There are limitations to our study. First, choice of rSnare and rTraction as a rescue method depended on individual preference of endoscopists, whereas patient background characteristics were adjusted using PSM. Second, follow-up of patients after endoscopic treatment was not standardized, making it difficult to determine the exact local recurrence rate in this study. Further prospective studies are needed to precisely determine the local recurrence rate associated with use of rescue techniques. Third, we carefully extracted lesions explicitly documented as experiencing intraoperative difficulty in the medical records. However, due to the retrospective nature of the study, the selection process was dependent on existing documentation. Whereas some eligible lesions may have been unintentionally excluded, we believe that PSM helped maintain consistency of results. Fourth, narrowing the focus to patients undergoing challenging colorectal ESD reduced the sample size from 1464 to 162 lesions, which may have introduced selection bias and limited generalizability of the findings. Although PSM further reduced the sample size, it was necessary to minimize confounding and ensure a balanced comparison between the groups. Key covariates were carefully selected using a DAG to avoid inappropriate adjustments for mediators. However, further prospective studies with larger sample sizes are needed to validate our findings and strengthen their applicability.

## Conclusions

In conclusion, use of a traction device as a rescue technique in difficult colorectal ESD resulted in a higher en bloc resection rate compared with converting to snare. However, utilizing the rTraction technique was associated with prolonged procedure time. Initiating the traction device within 75 minutes may aid in reducing the overall duration of difficult colorectal ESD.

## Supplementary Material

Supplementary Material Zusatzmaterial

## References

[1] Fujiya M Tanaka K Dokoshi T Efficacy and adverse events of EMR and endoscopic submucosal dissection for the treatment of colon neoplasms: a meta-analysis of studies comparing EMR and endoscopic submucosal dissection Gastrointest Endosc 2015 81 583 595 10.1016/j.gie.2014.07.034 25592748

[2] Yoshizaki T Toyonaga T Ikezawa N Tips for difficult colorectal endoscopic submucosal dissection Mini-invasive Surg 2022 6 17

[3] Sato K Ito S Kitagawa T Factors affecting the technical difficulty and clinical outcome of endoscopic submucosal dissection for colorectal tumors Surg Endosc 2014 28 2959 2965 10.1007/s00464-014-3558-y 24853849

[4] Hori K Uraoka T Harada K Predictive factors for technically difficult endoscopic submucosal dissection in the colorectum Endoscopy 2014 46 862 870 10.1055/s-0034-1377205 25208032

[5] Mizushima T Kato M Iwanaga I Technical difficulty according to location, and risk factors for perforation, in endoscopic submucosal dissection of colorectal tumors Surg Endosc 2015 29 133 139 10.1007/s00464-014-3665-9 24993172

[6] Pérez-Cuadrado-Robles E Snauwaert C Moreels T Risk factors for conversion to snare resection during colorectal endoscopic submucosal dissection in an expert Western center Endoscopy 2019 51 152 160 30206905 10.1055/a-0650-4562

[7] Okamoto K Muguruma N Kagemoto K Efficacy of hybrid endoscopic submucosal dissection (ESD) as a rescue treatment in difficult colorectal ESD cases Digest Endosc 2017 29 45 52 10.1111/den.12863 28425649

[8] Jinushi R Tashima T Terada R Effectiveness of a multi-loop traction device for colorectal endoscopic submucosal dissection performed by trainees: a pilot study Sci Rep 2022 12 10197 10.1038/s41598-022-14407-3 35715564 PMC9205909

[9] Dobashi A Storm AC Wong Kee Song LM An internal magnet traction device reduces procedure time for endoscopic submucosal dissection by expert and non-expert endoscopists: ex vivo study in a porcine colorectal model (with video) Surg Endosc 2019 33 2696 2703 31069502 10.1007/s00464-019-06817-8

[10] Yamasaki Y Takeuchi Y Uedo N Efficacy of traction-assisted colorectal endoscopic submucosal dissection using a clip-and-thread technique: A prospective randomized study Digest Endosc 2018 30 467 476 10.1111/den.13036 29424030

[11] Fujinami H Teramoto A Takahashi S Effectiveness of S-O clip-assisted colorectal endoscopic submucosal dissection J Clin Med 2021 11 141 10.3390/jcm11010141 35011881 PMC8745244

[12] Oyama T Counter traction makes endoscopic submucosal dissection easier Clin Endosc 2012 45 375 10.5946/ce.2012.45.4.375 23251884 PMC3521938

[13] Lopimpisuth C Simons M Akshintala VS Traction-assisted endoscopic submucosal dissection reduces procedure time and risk of serious adverse events: a systematic review and meta-analysis Surg Endosc 2022 36 1775 1788 10.1007/s00464-021-08452-8 33825013

[14] Su Y-F Cheng S-W Chang CC Efficacy and safety of traction-assisted endoscopic submucosal dissection: a meta-regression of randomized clinical trials Endoscopy 2020 52 338 348 10.1055/a-1106-3761 32110824

[15] Tanaka S Kashida H Saito Y JGES guidelines for colorectal endoscopic submucosal dissection/endoscopic mucosal resection Digest Endosc 2015 27 417 434 10.1111/den.12456 25652022

[16] Yamamoto K Michida T Nishida T Colorectal endoscopic submucosal dissection: Recent technical advances for safe and successful procedures Worl J Gastrointest Endosc 2015 7 1114 10.4253/wjge.v7.i14.1114 PMC4600177 26468335

[17] Suzuki Y Tanuma T Nojima M Comparison of dissection speed during colorectal ESD between the novel Multiloop (M-loop) traction method and ESD methods without traction Endosc Int Open 2020 08 E840 E847 10.1055/a-1161-8596 PMC7297616 32617388

[18] Yoshida N Naito Y Murakami T Tips for safety in endoscopic submucosal dissection for colorectal tumors Ann Transl Med 2017 5 185 185 10.21037/atm.2017.03.33 28616400 PMC5464937

[19] Imai K Hotta K Ito S A risk-prediction model for en bloc resection failure or perforation during endoscopic submucosal dissection of colorectal neoplasms Digest Endosc 2020 32 932 939 10.1007/s00464-019-06875-y 31883411

[20] Oh H-H Jung Y-W Jin B-C Predictive factors associated with technical difficulty in colorectal endoscopic submucosal dissection: A Honam Association for the Study of Intestinal Disease (HASID) multicenter study Medicine 2024 103 10.1097/MD.0000000000037936 PMC11049784 38669427

[21] Jeong YH Kim KO Park CS Risk factors of advanced adenoma in small and diminutive colorectal polyp J Korean Med Sci 2016 31 1426 10.3346/jkms.2016.31.9.1426 27510386 PMC4974184

[22] Agapov M Dvoinikova E Factors predicting clinical outcomes of endoscopic submucosal dissection in the rectum and sigmoid colon during the learning curve Endosc Int Open 2014 02 E235 E240 10.1055/s-0034-1377613 PMC4424868 26135099

[23] Gu F Jiang W Zhu J Risk factors for unsuccessful colorectal endoscopic submucosal dissection: A systematic review and meta-analysis Digest Liver Dis 2024 56 1288 1297 10.1016/j.dld.2023.11.030 38071178

[24] Chow CWS Fung TLD Chan PT Endoscopic submucosal dissection for colorectal polyps: outcome determining factors Surg Endosc 2023 37 1293 1302 10.1007/s00464-022-09672-2 36192659 PMC9529320

[25] Hayashi N Tanaka S Nishiyama S Predictors of incomplete resection and perforation associated with endoscopic submucosal dissection for colorectal tumors Gastrointest Endosc 2014 79 427 435 24210654 10.1016/j.gie.2013.09.014

[26] Imai K Hotta K Yamaguchi Y Preoperative indicators of failure of en bloc resection or perforation in colorectal endoscopic submucosal dissection: implications for lesion stratification by technical difficulties during stepwise training Gastrointest Endosc 2016 83 954 962 10.1016/j.gie.2015.08.024 26297870

[27] Ohata K Kobayashi N Sakai E Long-term outcomes after endoscopic submucosal dissection for large colorectal epithelial neoplasms: A prospective, multicenter, cohort trial from Japan Gastroenterology 2022 163 1423 143400 35810779 10.1053/j.gastro.2022.07.002

[28] Takamaru H Saito Y Yamada M Clinical impact of endoscopic clip closure of perforations during endoscopic submucosal dissection for colorectal tumors Gastrointest Endosc 2016 84 494 5020 26774353 10.1016/j.gie.2016.01.014

[29] Isomoto H Nishiyama H Yamaguchi N Clinicopathological factors associated with clinical outcomes of endoscopic submucosal dissection for colorectal epithelial neoplasms Endoscopy 2009 41 679 683 19670135 10.1055/s-0029-1214979

[30] Iacopini F Saito Y Bella A Colorectal endoscopic submucosal dissection: predictors and neoplasm-related gradients of difficulty Endosc Int Open 2017 05 E839 E846 10.1055/s-0043-113566 PMC5595579 28924587

[31] Winter K Kasprzyk P Nowicka Z Resection of early colorectal neoplasms using endoscopic submucosal dissection: A retrospective multicenter cohort study J Clin Med 2024 13 6989 10.3390/jcm13226989 39598133 PMC11595630

[32] McCarty TR Bazarbashi AN Thompson CC Hybrid endoscopic submucosal dissection (ESD) compared with conventional ESD for colorectal lesions: a systematic review and meta-analysis Endoscopy 2021 53 1048 1058 32947624 10.1055/a-1266-1855

[33] Milano RV Viale E Bartel MJ Resection outcomes and recurrence rates of endoscopic submucosal dissection (ESD) and hybrid ESD for colorectal tumors in a single Italian center Surg Endosc 2018 32 2328 2339 10.1007/s00464-017-5928-8 29098434

[34] Bae JH Yang D-H Lee S Optimized hybrid endoscopic submucosal dissection for colorectal tumors: a randomized controlled trial Gastrointest Endosc 2016 83 584 592 10.1016/j.gie.2015.06.057 26320696

[35] Ohata K Muramoto T Minato Y Usefulness of a multifunctional snare designed for colorectal hybrid endoscopic submucosal dissection (with video) Endosc Int Open 2018 06 E249 E253 10.1055/s-0043-124364 PMC5803000 29423435

[36] Kim YJ Kim ES Cho KB Comparison of clinical outcomes among different endoscopic resection methods for treating colorectal neoplasia Dig Dis Sci 2013 58 1727 1736 23385636 10.1007/s10620-013-2560-x

[37] Tamaru Y Kuwai T Miyakawa A Efficacy of a traction device for endoscopic submucosal dissection using a scissor-type knife: A randomized controlled trial Am J Gastroenterol 2022 117 1797 1804 10.14309/ajg.0000000000002019 36191269

