# Supplementary material for: Comparison of traction vs. snare as rescue methods for challenging colorectal endoscopic submucosal dissection: Propensity score-matched study
Source: Endosc Int Open. 2025 Mar 14;13:a25443279. doi: 10.1055/a-2544-3279 (PMC11922312; doi:10.1055/a-2544-3279)

Supplementary Table 1 Clinicopathological features of the rSnare and rTraction groups before propensity score matching.

	rSnare	rTraction	*P* value
Lesions, n	97	65	
Sex, n (%)			0.514
Male	55 (56.7)	41 (63.1)	
Female	42 (43.3)	24 (36.9)	
Age, years (mean ± SD)	70.4 (11.0)	68.5 (12.3)	0.31
Expert, n (%)	65 (67.0)	45 (69.2)	0.86
Location, n (%)			0.58
Right side of the colon	68 (70.1)	45 (69.2)	
Left side of the colon	13 (13.4)	12 (18.5)	
Rectum	16 (16.5)	8 (12.3)	
Morphology, n (%)			0.81
Protruded	19 (19.6)	10 (15.4)	
LST-G	32 (33.0)	22 (33.8)	
LST-NG	46 (47.4)	33 (50.8)	
Pathology, n (%)			0.046
adenocarcinoma	50 (51.5)	40 (61.5)	
adenoma	42 (43.3)	17 (26.2)	
SSL	5 (5.2)	8 (12.3)	
Fibrosis, n (%)	20 (20.6)	17 (26.2)	0.45
Invasion depth, n (%)			0.53
Submucosal invasion	8 (8.2)	3 (4.6)	
Tumor size, mm (mean ± SD)	20.8 (8.9)	28.3 (13.6)	< 0.001
Dissection speed, mm^2/min (SD)	5.6 (5.7)	5.9 (5.5)	0.76
En bloc resection, n (%)	51 (52.6)	62 (95.4)	< 0.001
R0 resection, n (%)	40 (41.2)	53 (81.5)	< 0.001
Procedure time, minutes (mean ± SD)	84.9 (53.2)	141.8 (78.8)	< 0.001
Intraoperative perforation, n (%)	6 (6.2)	2 (3.1)	0.48
Post-ESD bleeding, n (%)	5 (5.2)	3 (4.6)	> 0.99
Reasons for rescue, n (%)			< 0.05
Fibrosis	16 (16.5)	14 (21.5)	
Muscle injury or perforation	9 (9.3)	0 (0)	
Deep breathing or hyperperistalsis	17 (17.5)	3 (4.6)	
Poor scope operability	25 (25.8)	23 (35.4)	
Poor visibility of cutting line	30 (30.9)	25 (38.5)	

ESD, endoscopic submucosal dissection; LST-G, laterally spreading tumor, granular type; LST-NG, laterally spreading tumor, nongranular type; SD, standard deviation.

Supplementary Fig. 1 Study flow chart.

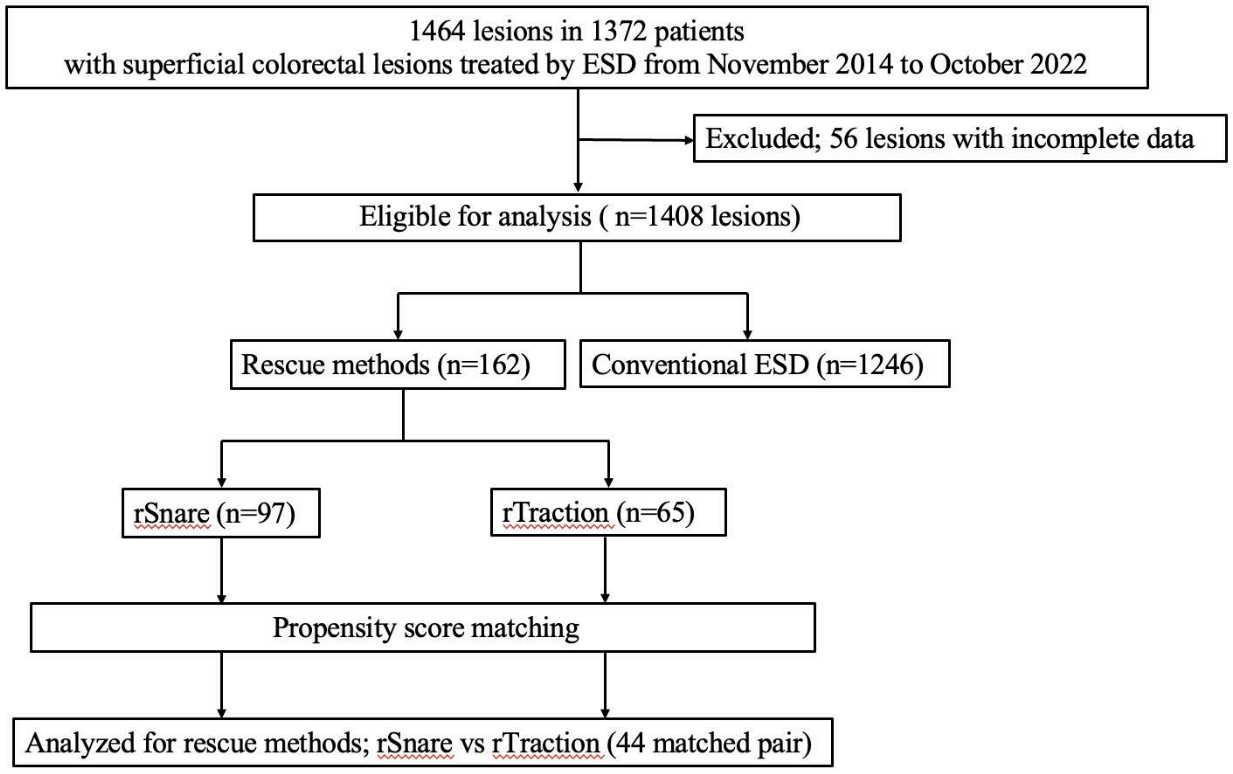

Supplementary Fig. 2 Directed acyclic graph representing causal relationships. The model identifies key confounders, including lesion characteristics (location, morphology, pathology, fibrosis, submucosal invasion, and tumor size) and technical factors (endoscopist experience and reasons for rescue). **a** In contrast, patient characteristics (sex and age) are not considered to have a direct impact on treatment allocation or outcomes. **b** The model illustrates that lesion characteristics (location, morphology, fibrosis, and tumor size), along with technical factors (traction device use) may directly affect procedure time.

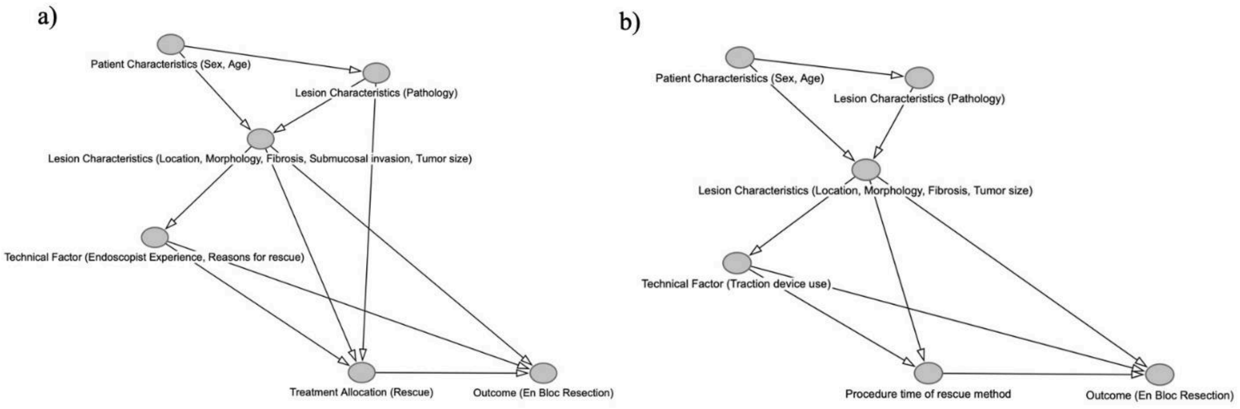

Supplement: Supplementary file 1 — Supplementary Material [file 10-1055-a-2544-3279_25482399.pdf]
